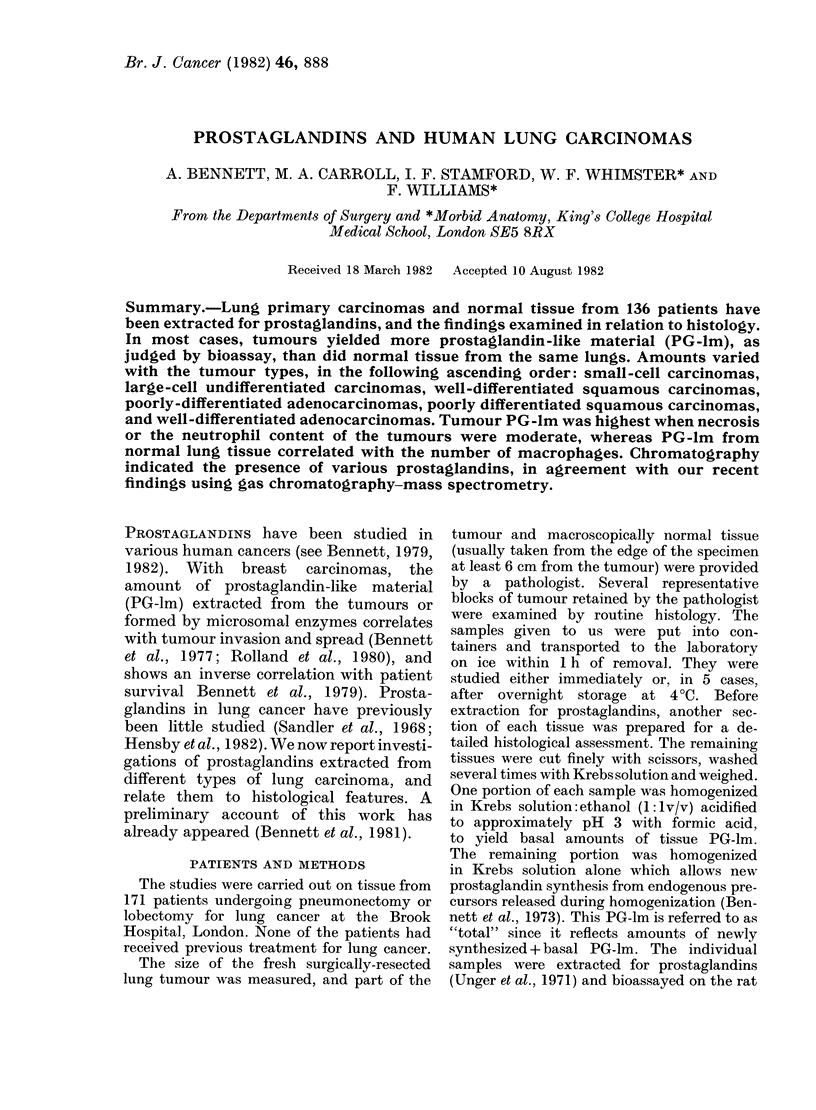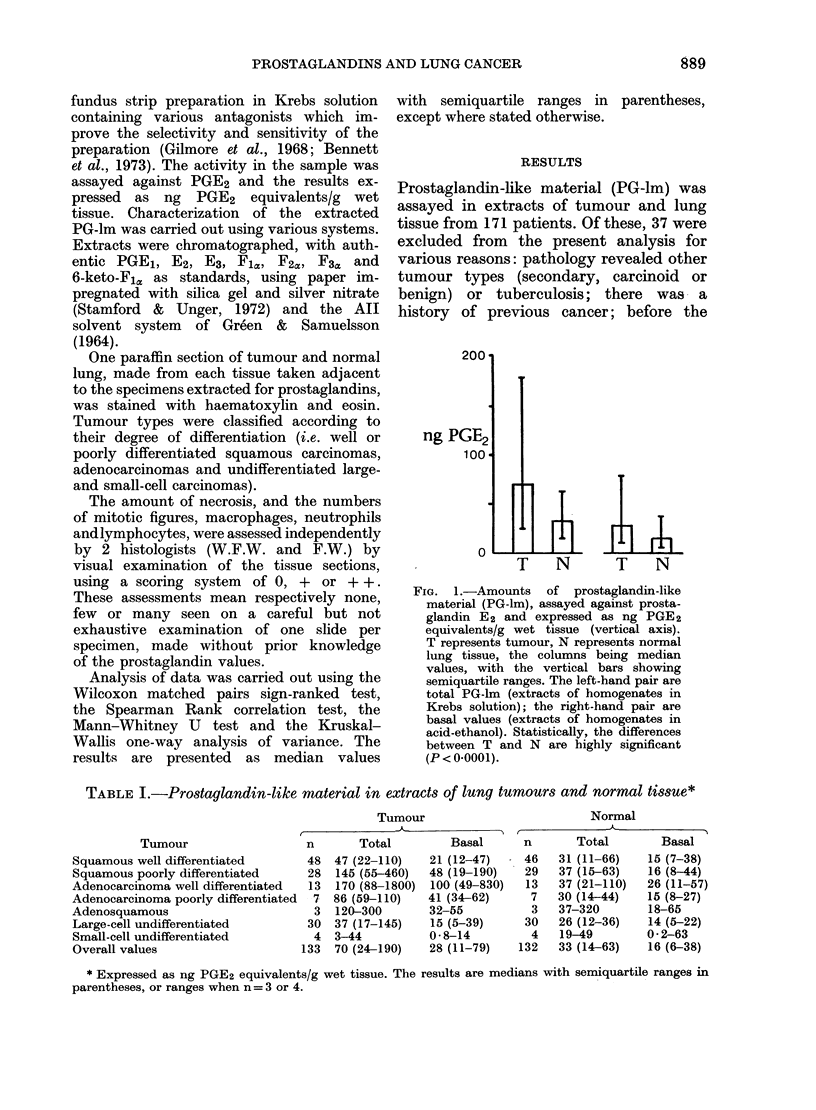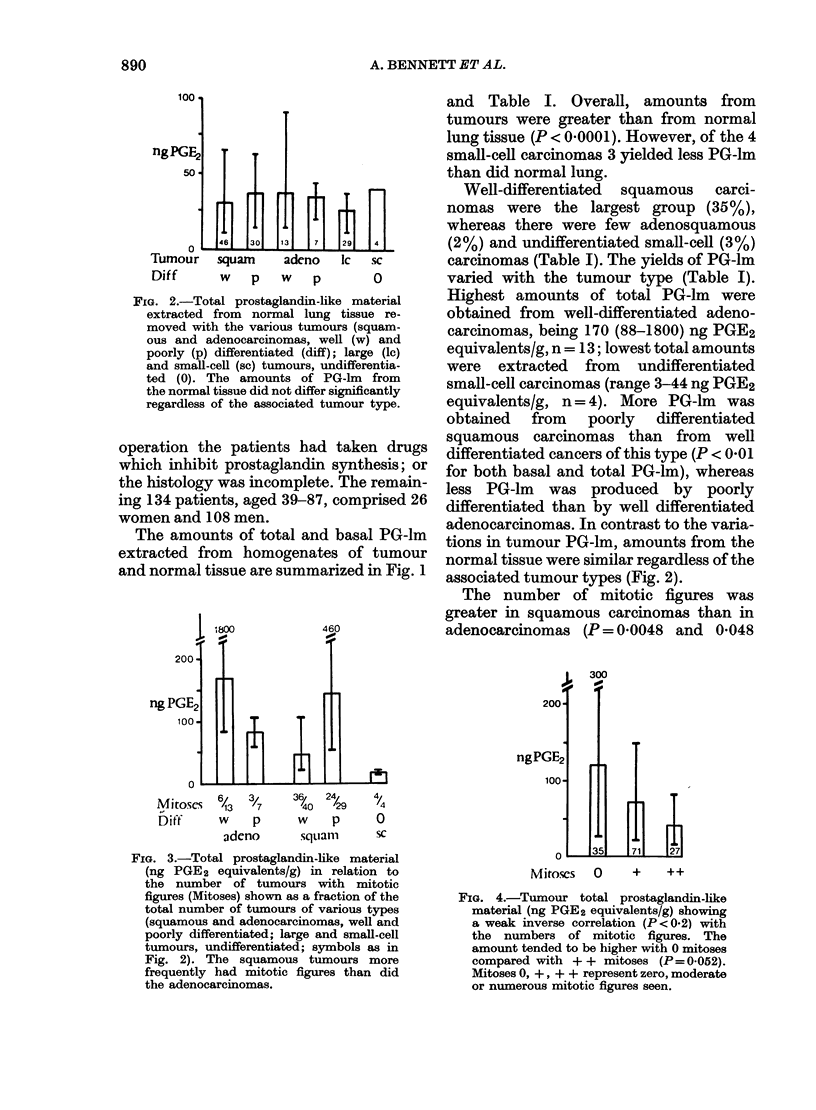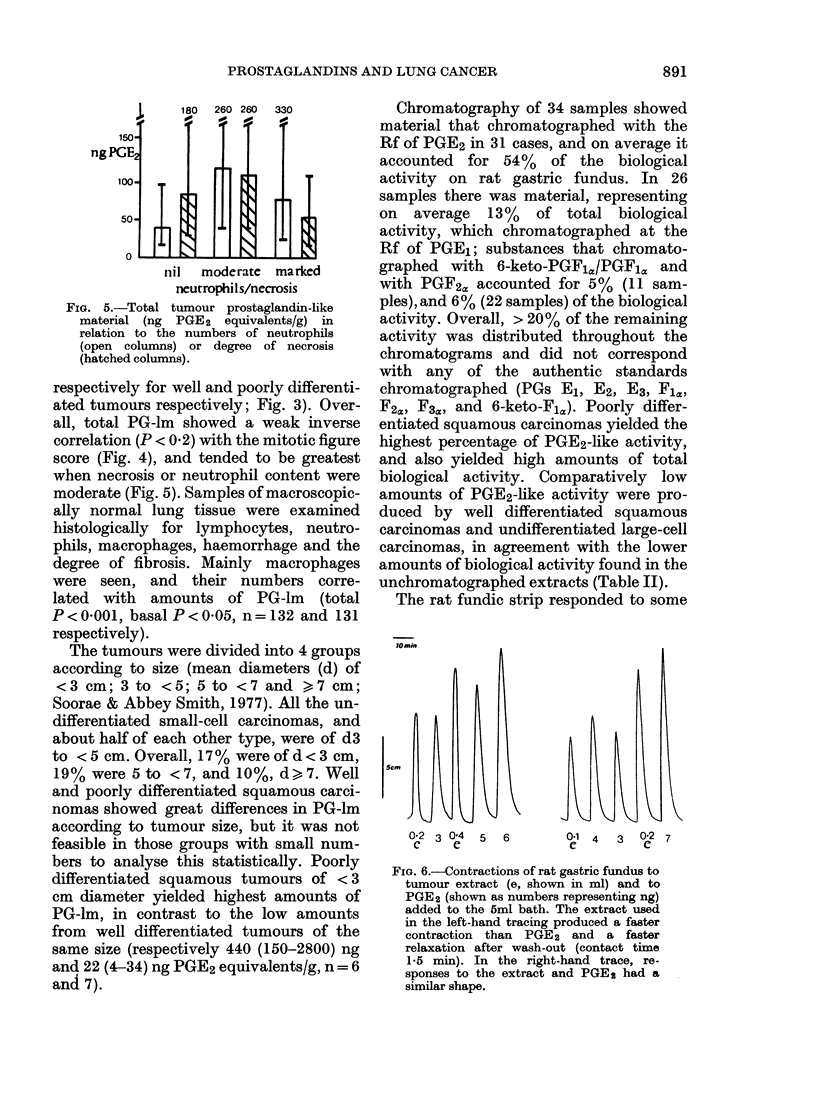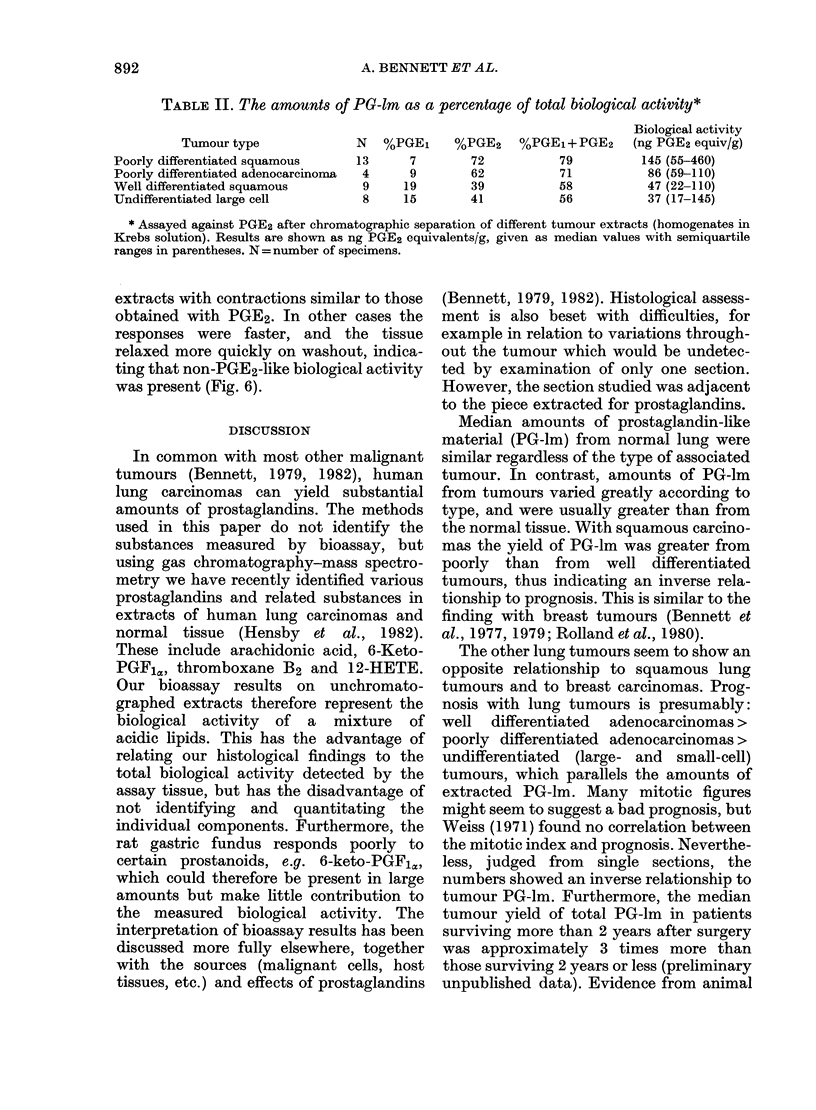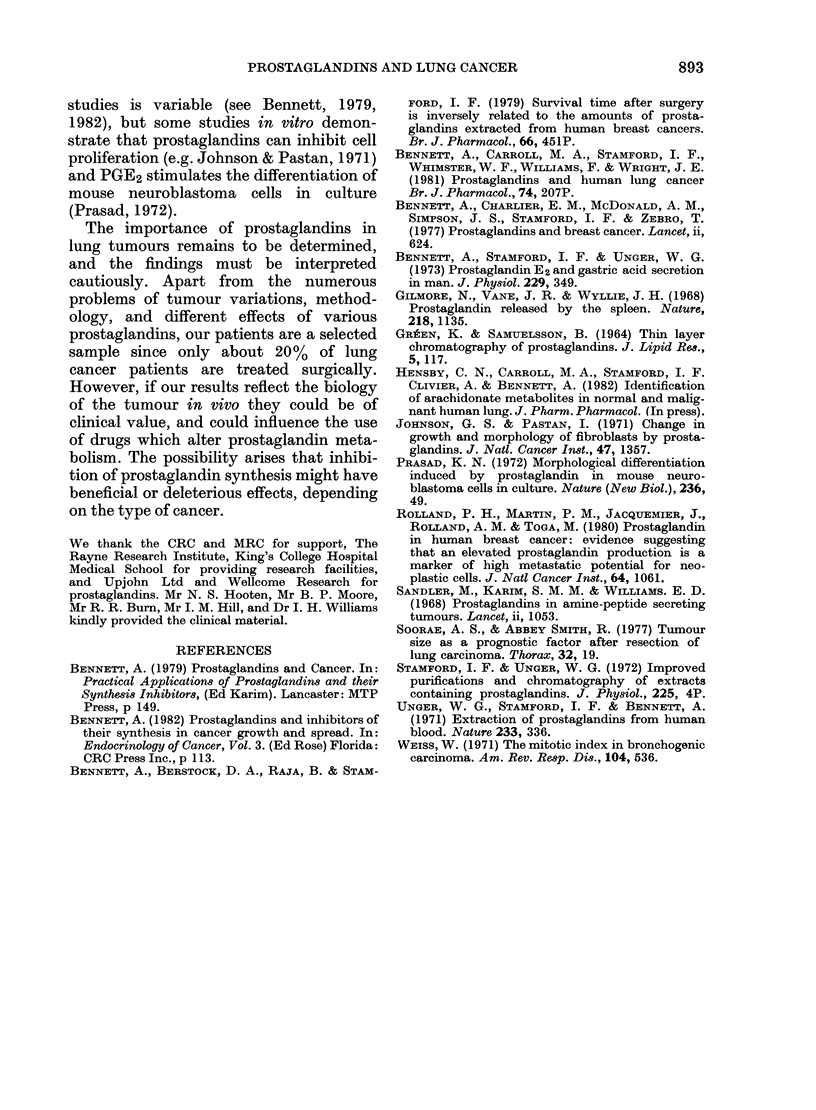# Prostaglandins and human lung carcinomas.

**DOI:** 10.1038/bjc.1982.298

**Published:** 1982-12

**Authors:** A. Bennett, M. A. Carroll, I. F. Stamford, W. F. Whimster, F. Williams

## Abstract

Lung primary carcinomas and normal tissue from 136 patients have been extracted for prostaglandins, and the findings examined in relation to histology. In most cases, tumours yielded more prostaglandin-like material (PG-lm), as judged by bioassay, than did normal tissue from the same lungs. Amounts varied with tumour types, in the following ascending order: small-cell carcinomas, large-cell undifferentiated carcinomas, well-differentiated squamous carcinomas, poorly-differentiated adenocarcinomas, poorly differentiated squamous carcinomas, and well-differentiated adenocarcinomas. Tumour PG-lm was highest when necrosis or the neutrophil content of the tumours were moderate, whereas PG-lm from normal lung tissue correlated with the number of macrophages. Chromatography indicated the presence of various prostaglandins, in agreement with our recent findings using gas chromatography--mass spectrometry.


Br. J. Cancer (1982) 46, 888

### PROSTAGLANDINS AND HUMAN LUNG CARCINOMAS

A. BENNETT, M. A. CARROLL, I. F. STAMFORD, W. F. WHIMSTER* AND F. WILLIAMS*

*From the Departments of Surgery and *Morbid Anatomy, King's College Hospital Medical School, London SE5 8RX*

Received 18 March 1982 Accepted 10 August 1982

**Summary.—Lung primary carcinomas and normal tissue from 136 patients have been extracted for prostaglandins, and the findings examined in relation to histology. In most cases, tumours yielded more prostaglandin-like material (PG-lm), as judged by bioassay, than did normal tissue from the same lungs. Amounts varied with the tumour types, in the following ascending order: small-cell carcinomas, large-cell undifferentiated carcinomas, well-differentiated squamous carcinomas, poorly-differentiated adenocarcinomas, poorly differentiated squamous carcinomas, and well-differentiated adenocarcinomas. Tumour PG-lm was highest when necrosis or the neutrophil content of the tumours were moderate, whereas PG-lm from normal lung tissue correlated with the number of macrophages. Chromatography indicated the presence of various prostaglandins, in agreement with our recent findings using gas chromatography–mass spectrometry.**

PROSTAGLANDINS have been studied in various human cancers (see Bennett, 1979, 1982). With breast carcinomas, the amount of prostaglandin-like material (PG-lm) extracted from the tumours or formed by microsomal enzymes correlates with tumour invasion and spread (Bennett *et al.*, 1977; Rolland *et al.*, 1980), and shows an inverse correlation with patient survival Bennett *et al.*, 1979). Prostaglandins in lung cancer have previously been little studied (Sandler *et al.*, 1968; Hensby *et al.*, 1982). We now report investigations of prostaglandins extracted from different types of lung carcinoma, and relate them to histological features. A preliminary account of this work has already appeared (Bennett *et al.*, 1981).

### PATIENTS AND METHODS

The studies were carried out on tissue from 171 patients undergoing pneumonectomy or lobectomy for lung cancer at the Brook Hospital, London. None of the patients had received previous treatment for lung cancer.

The size of the fresh surgically-resected lung tumour was measured, and part of the tumour and macroscopically normal tissue (usually taken from the edge of the specimen at least 6 cm from the tumour) were provided by a pathologist. Several representative blocks of tumour retained by the pathologist were examined by routine histology. The samples given to us were put into containers and transported to the laboratory on ice within 1 h of removal. They were studied either immediately or, in 5 cases, after overnight storage at 4°C. Before extraction for prostaglandins, another section of each tissue was prepared for a detailed histological assessment. The remaining tissues were cut finely with scissors, washed several times with Krebs solution and weighed. One portion of each sample was homogenized in Krebs solution:ethanol (1:1v/v) acidified to approximately pH 3 with formic acid, to yield basal amounts of tissue PG-lm. The remaining portion was homogenized in Krebs solution alone which allows new prostaglandin synthesis from endogenous precursors released during homogenization (Bennett *et al.*, 1973). This PG-lm is referred to as "total" since it reflects amounts of newly synthesized + basal PG-lm. The individual samples were extracted for prostaglandins (Unger *et al.*, 1971) and bioassayed on the rat fundus strip preparation in Krebs solution containing various antagonists which improve the selectivity and sensitivity of the preparation (Gilmore *et al.*, 1968; Bennett *et al.*, 1973). The activity in the sample was assayed against $PGE_2$ and the results expressed as ng $PGE_2$ equivalents/g wet tissue. Characterization of the extracted PG-lm was carried out using various systems. Extracts were chromatographed, with authentic $PGE_1$, $E_2$, $E_3$, $F_{1\alpha}$, $F_{2\alpha}$, $F_{3\alpha}$ and 6-keto-$F_{1\alpha}$ as standards, using paper impregnated with silica gel and silver nitrate (Stamford & Unger, 1972) and the AII solvent system of Gréen & Samuelsson (1964).

One paraffin section of tumour and normal lung, made from each tissue taken adjacent to the specimens extracted for prostaglandins, was stained with haematoxylin and eosin. Tumour types were classified according to their degree of differentiation (*i.e.* well or poorly differentiated squamous carcinomas, adenocarcinomas and undifferentiated large- and small-cell carcinomas).

The amount of necrosis, and the numbers of mitotic figures, macrophages, neutrophils and lymphocytes, were assessed independently by 2 histologists (W.F.W. and F.W.) by visual examination of the tissue sections, using a scoring system of 0, + or ++. These assessments mean respectively none, few or many seen on a careful but not exhaustive examination of one slide per specimen, made without prior knowledge of the prostaglandin values.

Analysis of data was carried out using the Wilcoxon matched pairs sign-ranked test, the Spearman Rank correlation test, the Mann–Whitney U test and the Kruskal–Wallis one-way analysis of variance. The results are presented as median values with semiquartile ranges in parentheses, except where stated otherwise.

### RESULTS

Prostaglandin-like material (PG-lm) was assayed in extracts of tumour and lung tissue from 171 patients. Of these, 37 were excluded from the present analysis for various reasons: pathology revealed other tumour types (secondary, carcinoid or benign) or tuberculosis; there was a history of previous cancer; before the

FIG. 1.—Amounts of prostaglandin-like material (PG-lm), assayed against prostaglandin $E_2$ and expressed as ng $PGE_2$ equivalents/g wet tissue (vertical axis). T represents tumour, N represents normal lung tissue, the columns being median values, with the vertical bars showing semiquartile ranges. The left-hand pair are total PG-lm (extracts of homogenates in Krebs solution); the right-hand pair are basal values (extracts of homogenates in acid-ethanol). Statistically, the differences between T and N are highly significant ($P < 0.0001$).

TABLE I.—*Prostaglandin-like material in extracts of lung tumours and normal tissue**

| | Tumour | | | Normal | | |
|---|---|---|---|---|---|---|
| Tumour | n | Total | Basal | n | Total | Basal |
| Squamous well differentiated | 48 | 47 (22–110) | 21 (12–47) | 46 | 31 (11–66) | 15 (7–38) |
| Squamous poorly differentiated | 28 | 145 (55–460) | 48 (19–190) | 29 | 37 (15–63) | 16 (8–44) |
| Adenocarcinoma well differentiated | 13 | 170 (88–1800) | 100 (49–830) | 13 | 37 (21–110) | 26 (11–57) |
| Adenocarcinoma poorly differentiated | 7 | 86 (59–110) | 41 (34–62) | 7 | 30 (14–44) | 15 (8–27) |
| Adenosquamous | 3 | 120–300 | 32–55 | 3 | 37–320 | 18–65 |
| Large-cell undifferentiated | 30 | 37 (17–145) | 15 (5–39) | 30 | 26 (12–36) | 14 (5–22) |
| Small-cell undifferentiated | 4 | 3–44 | 0·8–14 | 4 | 19–49 | 0·2–63 |
| Overall values | 133 | 70 (24–190) | 28 (11–79) | 132 | 33 (14–63) | 16 (6–38) |

* Expressed as ng $PGE_2$ equivalents/g wet tissue. The results are medians with semiquartile ranges in parentheses, or ranges when n = 3 or 4.

FIG. 2.—Total prostaglandin-like material extracted from normal lung tissue removed with the various tumours (squamous and adenocarcinomas, well (w) and poorly (p) differentiated (diff); large (lc) and small-cell (sc) tumours, undifferentiated (0). The amounts of PG-lm from the normal tissue did not differ significantly regardless of the associated tumour type.

operation the patients had taken drugs which inhibit prostaglandin synthesis; or the histology was incomplete. The remaining 134 patients, aged 39–87, comprised 26 women and 108 men.

The amounts of total and basal PG-lm extracted from homogenates of tumour and normal tissue are summarized in Fig. 1 and Table I. Overall, amounts from tumours were greater than from normal lung tissue ($P < 0{\cdot}0001$). However, of the 4 small-cell carcinomas 3 yielded less PG-lm than did normal lung.

Well-differentiated squamous carcinomas were the largest group (35%), whereas there were few adenosquamous (2%) and undifferentiated small-cell (3%) carcinomas (Table I). The yields of PG-lm varied with the tumour type (Table I). Highest amounts of total PG-lm were obtained from well-differentiated adenocarcinomas, being 170 (88–1800) ng $PGE_2$ equivalents/g, n = 13; lowest total amounts were extracted from undifferentiated small-cell carcinomas (range 3–44 ng $PGE_2$ equivalents/g, n = 4). More PG-lm was obtained from poorly differentiated squamous carcinomas than from well differentiated cancers of this type ($P < 0{\cdot}01$ for both basal and total PG-lm), whereas less PG-lm was produced by poorly differentiated than by well differentiated adenocarcinomas. In contrast to the variations in tumour PG-lm, amounts from the normal tissue were similar regardless of the associated tumour types (Fig. 2).

The number of mitotic figures was greater in squamous carcinomas than in adenocarcinomas ($P = 0{\cdot}0048$ and $0{\cdot}048$

FIG. 3.—Total prostaglandin-like material (ng $PGE_2$ equivalents/g) in relation to the number of tumours with mitotic figures (Mitoses) shown as a fraction of the total number of tumours of various types (squamous and adenocarcinomas, well and poorly differentiated; large and small-cell tumours, undifferentiated; symbols as in Fig. 2). The squamous tumours more frequently had mitotic figures than did the adenocarcinomas.

FIG. 4.—Tumour total prostaglandin-like material (ng $PGE_2$ equivalents/g) showing a weak inverse correlation ($P < 0{\cdot}2$) with the numbers of mitotic figures. The amount tended to be higher with 0 mitoses compared with + + mitoses ($P = 0{\cdot}052$). Mitoses 0, +, + + represent zero, moderate or numerous mitotic figures seen.

FIG. 5.—Total tumour prostaglandin-like material (ng $PGE_2$ equivalents/g) in relation to the numbers of neutrophils (open columns) or degree of necrosis (hatched columns).

respectively for well and poorly differentiated tumours respectively; Fig. 3). Overall, total PG-lm showed a weak inverse correlation ($P < 0{\cdot}2$) with the mitotic figure score (Fig. 4), and tended to be greatest when necrosis or neutrophil content were moderate (Fig. 5). Samples of macroscopically normal lung tissue were examined histologically for lymphocytes, neutrophils, macrophages, haemorrhage and the degree of fibrosis. Mainly macrophages were seen, and their numbers correlated with amounts of PG-lm (total $P < 0{\cdot}001$, basal $P < 0{\cdot}05$, n = 132 and 131 respectively).

The tumours were divided into 4 groups according to size (mean diameters (d) of < 3 cm; 3 to < 5; 5 to < 7 and ≥ 7 cm; Soorae & Abbey Smith, 1977). All the undifferentiated small-cell carcinomas, and about half of each other type, were of d3 to < 5 cm. Overall, 17% were of d < 3 cm, 19% were 5 to < 7, and 10%, d ≥ 7. Well and poorly differentiated squamous carcinomas showed great differences in PG-lm according to tumour size, but it was not feasible in those groups with small numbers to analyse this statistically. Poorly differentiated squamous tumours of < 3 cm diameter yielded highest amounts of PG-lm, in contrast to the low amounts from well differentiated tumours of the same size (respectively 440 (150–2800) ng and 22 (4–34) ng $PGE_2$ equivalents/g, n = 6 and 7).

Chromatography of 34 samples showed material that chromatographed with the Rf of $PGE_2$ in 31 cases, and on average it accounted for 54% of the biological activity on rat gastric fundus. In 26 samples there was material, representing on average 13% of total biological activity, which chromatographed at the Rf of $PGE_1$; substances that chromatographed with 6-keto-$PGF_{1\alpha}$/$PGF_{1\alpha}$ and with $PGF_{2\alpha}$ accounted for 5% (11 samples), and 6% (22 samples) of the biological activity. Overall, > 20% of the remaining activity was distributed throughout the chromatograms and did not correspond with any of the authentic standards chromatographed (PGs $E_1$, $E_2$, $E_3$, $F_{1\alpha}$, $F_{2\alpha}$, $F_{3\alpha}$, and 6-keto-$F_{1\alpha}$). Poorly differentiated squamous carcinomas yielded the highest percentage of $PGE_2$-like activity, and also yielded high amounts of total biological activity. Comparatively low amounts of $PGE_2$-like activity were produced by well differentiated squamous carcinomas and undifferentiated large-cell carcinomas, in agreement with the lower amounts of biological activity found in the unchromatographed extracts (Table II).

The rat fundic strip responded to some

FIG. 6.—Contractions of rat gastric fundus to tumour extract (e, shown in ml) and to $PGE_2$ (shown as numbers representing ng) added to the 5ml bath. The extract used in the left-hand tracing produced a faster contraction than $PGE_2$ and a faster relaxation after wash-out (contact time 1·5 min). In the right-hand trace, responses to the extract and $PGE_2$ had a similar shape.

TABLE II. *The amounts of PG-lm as a percentage of total biological activity**

| Tumour type | N | %$PGE_1$ | %$PGE_2$ | %$PGE_1+PGE_2$ | Biological activity (ng $PGE_2$ equiv/g) |
|---|---|---|---|---|---|
| Poorly differentiated squamous | 13 | 7 | 72 | 79 | 145 (55–460) |
| Poorly differentiated adenocarcinoma | 4 | 9 | 62 | 71 | 86 (59–110) |
| Well differentiated squamous | 9 | 19 | 39 | 58 | 47 (22–110) |
| Undifferentiated large cell | 8 | 15 | 41 | 56 | 37 (17–145) |

* Assayed against $PGE_2$ after chromatographic separation of different tumour extracts (homogenates in Krebs solution). Results are shown as ng $PGE_2$ equivalents/g, given as median values with semiquartile ranges in parentheses. N = number of specimens.

extracts with contractions similar to those obtained with $PGE_2$. In other cases the responses were faster, and the tissue relaxed more quickly on washout, indicating that non-$PGE_2$-like biological activity was present (Fig. 6).

### DISCUSSION

In common with most other malignant tumours (Bennett, 1979, 1982), human lung carcinomas can yield substantial amounts of prostaglandins. The methods used in this paper do not identify the substances measured by bioassay, but using gas chromatography–mass spectrometry we have recently identified various prostaglandins and related substances in extracts of human lung carcinomas and normal tissue (Hensby *et al.*, 1982). These include arachidonic acid, 6-Keto-$PGF_{1\alpha}$, thromboxane $B_2$ and 12-HETE. Our bioassay results on unchromatographed extracts therefore represent the biological activity of a mixture of acidic lipids. This has the advantage of relating our histological findings to the total biological activity detected by the assay tissue, but has the disadvantage of not identifying and quantitating the individual components. Furthermore, the rat gastric fundus responds poorly to certain prostanoids, *e.g.* 6-keto-$PGF_{1\alpha}$, which could therefore be present in large amounts but make little contribution to the measured biological activity. The interpretation of bioassay results has been discussed more fully elsewhere, together with the sources (malignant cells, host tissues, etc.) and effects of prostaglandins (Bennett, 1979, 1982). Histological assessment is also beset with difficulties, for example in relation to variations throughout the tumour which would be undetected by examination of only one section. However, the section studied was adjacent to the piece extracted for prostaglandins.

Median amounts of prostaglandin-like material (PG-lm) from normal lung were similar regardless of the type of associated tumour. In contrast, amounts of PG-lm from tumours varied greatly according to type, and were usually greater than from the normal tissue. With squamous carcinomas the yield of PG-lm was greater from poorly than from well differentiated tumours, thus indicating an inverse relationship to prognosis. This is similar to the finding with breast tumours (Bennett *et al.*, 1977, 1979; Rolland *et al.*, 1980).

The other lung tumours seem to show an opposite relationship to squamous lung tumours and to breast carcinomas. Prognosis with lung tumours is presumably: well differentiated adenocarcinomas > poorly differentiated adenocarcinomas > undifferentiated (large- and small-cell) tumours, which parallels the amounts of extracted PG-lm. Many mitotic figures might seem to suggest a bad prognosis, but Weiss (1971) found no correlation between the mitotic index and prognosis. Nevertheless, judged from single sections, the numbers showed an inverse relationship to tumour PG-lm. Furthermore, the median tumour yield of total PG-lm in patients surviving more than 2 years after surgery was approximately 3 times more than those surviving 2 years or less (preliminary unpublished data). Evidence from animal studies is variable (see Bennett, 1979, 1982), but some studies *in vitro* demonstrate that prostaglandins can inhibit cell proliferation (e.g. Johnson & Pastan, 1971) and $PGE_2$ stimulates the differentiation of mouse neuroblastoma cells in culture (Prasad, 1972).

The importance of prostaglandins in lung tumours remains to be determined, and the findings must be interpreted cautiously. Apart from the numerous problems of tumour variations, methodology, and different effects of various prostaglandins, our patients are a selected sample since only about 20% of lung cancer patients are treated surgically. However, if our results reflect the biology of the tumour *in vivo* they could be of clinical value, and could influence the use of drugs which alter prostaglandin metabolism. The possibility arises that inhibition of prostaglandin synthesis might have beneficial or deleterious effects, depending on the type of cancer.

We thank the CRC and MRC for support, The Rayne Research Institute, King's College Hospital Medical School for providing research facilities, and Upjohn Ltd and Wellcome Research for prostaglandins. Mr N. S. Hooten, Mr B. P. Moore, Mr R. R. Burn, Mr I. M. Hill, and Dr I. H. Williams kindly provided the clinical material.

### REFERENCES

BENNETT, A. (1979) Prostaglandins and Cancer. In: *Practical Applications of Prostaglandins and their Synthesis Inhibitors*, (Ed Karim). Lancaster: MTP Press, p 149.

BENNETT, A. (1982) Prostaglandins and inhibitors of their synthesis in cancer growth and spread. In: *Endocrinology of Cancer, Vol.* 3. (Ed Rose) Florida: CRC Press Inc., p 113.

BENNETT, A., BERSTOCK, D. A., RAJA, B. & STAMFORD, I. F. (1979) Survival time after surgery is inversely related to the amounts of prostaglandins extracted from human breast cancers. *Br. J. Pharmacol.*, **66**, 451P.

BENNETT, A., CARROLL, M. A., STAMFORD, I. F., WHIMSTER, W. F., WILLIAMS, F. & WRIGHT, J. E. (1981) Prostaglandins and human lung cancer *Br. J. Pharmacol.*, **74**, 207P.

BENNETT, A., CHARLIER, E. M., MCDONALD, A. M., SIMPSON, J. S., STAMFORD, I. F. & ZEBRO, T. (1977) Prostaglandins and breast cancer. *Lancet*, ii, 624.

BENNETT, A., STAMFORD, I. F. & UNGER, W. G. (1973) Prostaglandin $E_2$ and gastric acid secretion in man. *J. Physiol.* **229**, 349.

GILMORE, N., VANE, J. R. & WYLLIE, J. H. (1968) Prostaglandin released by the spleen. *Nature*, **218**, 1135.

GRÉEN, K. & SAMUELSSON, B. (1964) Thin layer chromatography of prostaglandins. *J. Lipid Res.*, **5**, 117.

HENSBY, C. N., CARROLL, M. A., STAMFORD, I. F. CLIVIER, A. & BENNETT, A. (1982) Identification of arachidonate metabolites in normal and malignant human lung. *J. Pharm. Pharmacol.* (In press).

JOHNSON, G. S. & PASTAN, I. (1971) Change in growth and morphology of fibroblasts by prostaglandins. *J. Natl. Cancer Inst.*, **47**, 1357.

PRASAD, K. N. (1972) Morphological differentiation induced by prostaglandin in mouse neuroblastoma cells in culture. *Nature* (*New Biol.*), **236**, 49.

ROLLAND, P. H., MARTIN, P. M., JACQUEMIER, J., ROLLAND, A. M. & TOGA, M. (1980) Prostaglandin in human breast cancer: evidence suggesting that an elevated prostaglandin production is a marker of high metastatic potential for neoplastic cells. *J. Natl Cancer Inst.*, **64**, 1061.

SANDLER, M., KARIM, S. M. M. & WILLIAMS. E. D. (1968) Prostaglandins in amine-peptide secreting tumours. *Lancet*, ii, 1053.

SOORAE, A. S., & ABBEY SMITH, R. (1977) Tumour size as a prognostic factor after resection of lung carcinoma. *Thorax*, **32**, 19.

STAMFORD, I. F. & UNGER, W. G. (1972) Improved purifications and chromatography of extracts containing prostaglandins. *J. Physiol.*, **225**, 4P.

UNGER, W. G., STAMFORD, I. F. & BENNETT, A. (1971) Extraction of prostaglandins from human blood. *Nature* **233**, 336.

WEISS, W. (1971) The mitotic index in bronchogenic carcinoma. *Am. Rev. Resp. Dis.*, **104**, 536.